# Proximate, Elemental, and Functional Properties of Novel Solid Dispersions of *Moringa oleifera* Leaf Powder

**DOI:** 10.3390/molecules27154935

**Published:** 2022-08-03

**Authors:** Nontsikelelo Noxolo Tafu, Victoria A. Jideani

**Affiliations:** Department of Food Science & Technology, Cape Peninsula University of Technology, Bellville 7535, South Africa; tafunoxolo@gmail.com

**Keywords:** solid dispersions, *Moringa oleifera* leaf powder, nutritional properties, functional foods, functional properties, solubility, elemental composition, EDS

## Abstract

*Moringa oleifera* leaf powder (MOLP) is a rich source of antioxidants, protein, minerals, vitamins, and various phytochemicals and has been used to combat malnutrition in many countries. However, despite its many benefits, MOLP has low a solubility in water, necessitating the development of ways to address this issue. To improve the solubility of MOLP, solid-dispersed *Moringa oleifera* leaf powders (SDMOLPs) have been developed through freeze-drying, melting, microwave irradiation, and solvent evaporation methods using polyethylene glycols (PEG4000 and PEG6000) (1:1) as hydrophilic carriers. The solid dispersions were evaluated for their proximate composition using standard analytical procedures. Elemental composition was characterized using scanning electron microscopy (SEM) with energy-dispersive X-ray spectroscopy (EDS). Water absorption capacity (WAC) and water-solubility were further evaluated as functional properties. Proximate composition revealed that MOLP and SDMOLPs were rich in protein, energy, carbohydrate, ash, and fat contents. MOLP solid dispersions are a major source of minerals (Ca, Mg, Cu, and Zn), and can be used to alleviate many mineral deficiencies. All solid dispersions had significantly higher (*p* < 0.05) solubilities (ranging from 54 to 64%) and WAC (ranging from 468.86 to 686.37%), relative to that of pure MOLP. The increased solubility of SDMOLPs may be attributed to the hydrogen bonds and intermolecular interactions between MOLP and the hydrophilic carriers. The results indicate that the solid dispersion technique can be successfully employed to improve the solubility of MOLP. And the solid-dispersed MOLPs with enhanced functional properties may be useful as functional ingredients in foods and beverages, dietary supplements, or nutraceutical formulations.

## 1. Introduction

The *Moringa oleifera* crop, which grows well in the humid tropics or scorching drylands can thrive on less fertile soils and is drought resistant. Moringa (*Moringa oleifera Lam*.) is often known as “The Miracle Tree” because of its nutritional benefits, medical properties, and ability to conserve the environment. Moringa is regarded as one of the world’s most useful trees since nearly every part of the Moringa tree can be utilized for food, medicine, or industry [1]. This tree has the potential to raise food security, improve nutrition, and promote rural development. Several studies have shown that the leaves of this plant are extremely nutritious [2,3,4,5], and can be used to combat malnutrition, particularly for nursing mothers and infants because of the high protein (20–35%), carbohydrates (35–48%), fat (4–7%) in the leaves [6]. *Moringa oleifera* leaves contain a rich concentration of multi-vitamins (A, B, C and E), micro and macro minerals (Ca, K, Mg, P, S, Mn, Fe, Zn, and Cu), fatty acids, phenolics, antioxidants, and essential amino acids for body development [4,7]. The fresh leaves are eaten as greens, salads, vegetable curries, pickles, and seasoning. The dried leaves are crushed or pound and sifted into leaf powder, which can be added to sauces and foods as a condiment. MOLP has been used in green teas, amahewu, soups, spices, yogurt, capsules, bread, and biscuits, among others, to add functionality to other foods through fortification and supplementation. Moringa leaves protein rival that of milk and eggs [8,9]. These leaves also have medicinal properties and health benefits. Moringa has been used to treat more than 300 conditions due to its antioxidant, anticancer, anti-inflammatory, antidiabetic, antifungal, antidepressant, and antimicrobial properties, among others [10]. 

While the vast potential of *Moringa oleifera* leaf powder (MOLP) has been reported, MOLP is still largely underutilized as a functional ingredient in food systems. The major problem associated with MOLP is its inadequate solubility in liquid systems [4,7], which ultimately limits its utility in the food and beverage industry [4]. Solubility is a crucial requirement for a high protein ingredient to be an effective nutritional ingredient in high moisture food applications such as protein-enriched beverages. It is therefore important to develop an appropriate formulation to improve the solubility of MOLP. Solubility enhancement technologies are an integral part of ingredient applications and have been well-studied in both food and pharmaceutical applications. Several methods have been developed to enhance the solubility of poorly water-soluble ingredients and compounds including solid dispersion [11,12,13,14,15,16,17,18,19], micronization [18,20], complexation with cyclodextrins, nanonization, solubilization using co-solvents, salt formation, and milling [4]. Although each of these strategies has been used to enhance the dissolution and solubility of poorly water-soluble bioactive compounds and ingredients, each has its substantial limitations, ranging from long preparation times to particle agglomeration and poor flow properties [21]. Solid dispersion, however, remains one of the most popular and successful methods for enhancing the solubility of products because of its simplicity (easier to produce and more applicable) and efficiency. In addition to increasing solubility, the solid dispersion technique can be used to solve various problems such as dissolution, stability, and bioavailability [15,22,23]. Lan et al. [24] discovered that the solid dispersion technique can also mitigate off flavors or the bitter taste of plant compounds. Moreover, Tafu and Jideani [22], reported that the technique could increase the thermal stability of poorly stable ingredients, allowing them to be employed in high thermal processing systems. To improve the solubility and dissolution of poorly soluble ingredients from solid dispersions, the particle size should be reduced, crystalline ingredients should become amorphous, solid solutions should form, complexes should form, aggregation and agglomeration should be reduced, the ingredient should wet better, and the ingredient should be solubilized by the carrier [21,22].

The solid dispersion technique is a promising means that has been used to improve the solubility of plant-based ingredients such as beta carotene [23] and pea protein. The fundamental concept behind this technique is to disperse poorly water-soluble ingredients in a hydrophilic carrier matrix using spray-drying, solvent evaporation, kneading, microwave irradiation, freeze-drying, and/or melting techniques to obtain a dispersed state with increased solubility [25]. In the melting method, the ingredients and carrier are mixed and heated directly until they melt. After melting, the mixture is solidified. The solvent evaporation method includes dissolving the component and carrier in a common solvent, followed by solvent removal to generate the solid mass. Nevertheless, the solvent evaporation method is common in solid dispersions. 

The solid dispersion technique is usually employed with polymeric carriers such as polyethylene glycol (PEG) derivatives, polyvinylpyrrolidone (PVP), sugars, and cyclodextrins, among others [26,27,28,29]. PEGs are widely employed in the food industry and pharmaceutical industries as food additives, carriers, and solubilizing excipients because of their low melting point (55–63 °C), wide compatibility, hydrophilicity, the ability to dissolve rapidly, and the ability to dissolve in many organic solvents [30]. PEG, as a hydrophilic carrier, is commonly used in the preparation of solid dispersion formulations and is available in a range of molecular weights (1500–20,000). Each polymer molecular weight has distinct properties that can result in a wide range of solid dispersion formulation properties. Studies on the development of herbal products using the solid dispersion technique are increasing. However, solid dispersion containing MOLP is not available to date. Therefore, the objective of this study was to develop MOLP-PEG solid dispersions by utilizing several approaches (freeze-drying, melting, solvent evaporation, and microwave irradiation).

## 2. Materials and Methods

### 2.1. Source of Materials 

*Moringa oleifera* leaf powder (MOLP) was purchased from Supa Nutri PTY Ltd. in Cape Town, South Africa. The polyethylene glycols were supplied by Spellbound Laboratory Solutions CC in Cape Town, South Africa. Analytical grade chemical reagents were used.

### 2.2. Preparation of Solid Dispersions 

Four alternative procedures (solvent evaporation, freeze-drying, melting, and microwave irradiation) were employed to create solid dispersions of MOLP utilizing PEG4000 and PEG6000 carriers, respectively. 

#### 2.2.1. Solvent Evaporation Method 

Solid dispersion was prepared by solvent evaporation using the methods in [22,31], with minor modifications. The MOLP and PEGs were carefully weighed in equal parts (50 g) at a 1:1 ratio. The MOLP and carrier were dissolved in appropriate amounts of absolute methanol (60 mL) and stirred continuously at 200 rpm for 20 min using magnetic stirring. To obtain a dry cake mass, the solvent was rapidly evaporated in an air oven at 50 °C for 24 h with mild heating and then stored at room temperature for 48–72 h in a desiccator. The obtained solid dispersion was triturated and sieved using a 125 µm sieve. The products were placed in a desiccator until further usage.

#### 2.2.2. Freeze-Drying Technique

The freeze-drying method, which was modified from [22,32], was employed. The MOLP and PEG were accurately weighed (50 g each). The MOLP was dissolved in 5 mL of 96% ethanol, and the PEG in 70 mL of distilled water. On a magnetic stirrer, the solution was mixed and then homogenized by gradually adding the MOLP-containing beaker to the PEG-containing beaker while stirring at 500 rpm for 30 min. The homogenized mixture was frozen in an ultra-freezer at a temperature of −70 °C. A freeze-dryer (Bench Top Pro 3L ES-55, SP Scientific, Ipswich, UK) was then used to dry the frozen form for two days at 50 °C. Solid dispersions were triturated and filtered through a 150–125 µm sieve before being deposited in centrifuge tubes and stored in a desiccator for subsequent analysis. 

#### 2.2.3. Melting Method Technique

The melting process described by Tafu and Jideani [22,33] was adopted, with minor changes. Separately, 50 g (1:1) MOLP and PEG were properly weighed into 400 mL glass beakers. The carrier was first melted using a magnetic stirrer at 58 °C for 10 min before adding the MOLP to the molten base to achieve a molecular dispersion. The molten liquid was properly mixed and stirred for 30 min. The molten mixture was rapidly cooled and solidified in an ice bath while being constantly stirred. After solidification, the solid material was crushed and filtered over a 150–125 µm sieve. The resulting product was stored in centrifuge tubes and kept in a desiccator until use. 

#### 2.2.4. Microwave Method Technique

The microwave irradiation method was modified from [12,28,34]. A total of 50 g of MOLP and PEG was mixed 1:1 in a 400 mL glass beaker and microwaved for 2 min at 360 W in a domestic microwave (LG model). The solid dispersion obtained from this method was cooled and pulverized with a mortar and pestle before being passed through a 150–125 µm sieve. The samples were kept in centrifuge tubes at room temperature (20 °C) until further examination.

### 2.3. Characterization of MOLP and Solid-Dispersed MOLPs

#### 2.3.1. Proximal Analyses

The moisture and ash content in pure MOLP and SDMOLP were determined using a moisture content analyzer (Denver instrument IR-30) and muffle furnace method, respectively [35]. The protein content was determined using a nitrogen analyzer (Leco-TruSpec-N) furnace set at 950 °C, and the protein factor used was 6.25. The fat content was determined using a Soxhlet system. The carbohydrates were determined by difference (Equation (1)) [35].

%Carbohydrate = 100 − (%moisture + %fat + %ash + %crude protein)(1)

The caloric value (Kcal) of the sample was determined using the “Atwater factor” by multiplying the values of the crude protein, fat, and carbohydrate by 3.99, 9.1, and 3.99, respectively, and summing the results [6]. 

#### 2.3.2. Elemental Analysis

SEM-energy dispersive X-ray spectroscopy (SEM-EDS) with an EDS Oxford Instruments^®^ XMax 20 mm2 detector (Carl Zeiss Microscopy, Oberkochen, Germany) was used to analyze the morphology and the elemental composition of the pure MOLP and solid-dispersed MOLPs. Operating conditions of 20 V accelerating voltage and 8.5 mm were applied. The mineral phases were studied using a Philips diffractometer (PW1840), HT generator (PW1729), and chart recorder (PW8203A) (GmbH, Germany). 

#### 2.3.3. Functional Properties of MOLP and Solid-Dispersed MOLP

##### Determination of Water Absorption Capacity

The water absorption capacity (WAC) was determined using a method described by Köhn et al. [34] and Sultana [6] with some modifications. About 3 g of a sample (MOLP, solid dispersions) was weighed into pre-weighed graduated 50 mL conical centrifuge tubes and mixed with 30 mL of distilled water with continuous stirring for 5 min. The suspension was allowed to stand at 30 ± 2 °C for an hour. The resulting slurry was centrifuged at 3000 rpm for 5 min. Thereafter, the supernatant was decanted, and the water retained per gram sample was calculated using Equation (2).

(2) $$\mathrm{WAC}\;(\%)=\frac{\mathrm{Weight}\;\mathrm{of}\;\mathrm{bound}\;\mathrm{water}\;\left(g\right)}{\mathrm{Weight}\;\mathrm{of}\;\mathrm{sample}\;\left(g\right)}\;\times \;100$$

##### Determination of Solubility

The solubility of pure MOLP and solid-dispersed MOLP was determined by slightly modifying the method of Itagi et al. [36]. A sample equivalent to 3 g was accurately weighed and dispersed in 30 mL of distilled water in a 50 mL centrifuge tube. The solution was mixed at a high-speed of 1000 rpm for 20 min using a vortex. The dispersed powder was then centrifuged at 3000 rpm for 5 min. The supernatant was further filtered using a 0.45 µm filter membrane and aliquots of the supernatant were carefully pipetted and transferred to a pre-weighed porcelain dish and then oven-dried at 105 °C in an air oven for 5 h. The solubility (%) of the samples was therefore calculated by taking the weighted difference using Equation (3).

(3) $$\mathrm{Solubility}\;(g/100g)=\frac{\mathrm{Weight}\;\mathrm{of}\;\mathrm{dried}\;\mathrm{sample}\;\mathrm{and}\;\mathrm{dish}-\mathrm{weight}\;\mathrm{of}\;\mathrm{the}\;\mathrm{dish}\;}{\mathrm{Weight}\;\mathrm{of}\;\mathrm{powder}\;\mathrm{sample}}\;\times \;100$$

### 2.4. Data Analysis 

Multivariate analysis of variance (MANOVA) was used to evaluate the significant differences (*p* < 0.05) in characteristics between the samples. Duncan’s multiple range test was used to distinguish means if there was a significant difference (IBM SPSS version 26, 2020).

## 3. Results and Discussions

### 3.1. Nutritional Compositions of MOLP and Solid-Dispersed MOLPs

#### 3.1.1. Moisture Content

The proximate composition for MOLP and SDMOLPs is presented in Table 1. The average moisture content of MOLP was 8.44%. The observed moisture value reported in this study is in agreement with the value (8.20%) reported by Okiki et al [37], but lower compared to the 10% recommended for dried vegetables. The solid dispersions (SDMOLP) revealed a moisture content that ranged from 2.65 to 4.03%. The lowest moisture content (2.65%) was recorded for solvent evaporation PEG6000 solid dispersion, while the highest value of 4.03% was observed in the freeze-dried PEG4000 solid dispersion. A significant difference (*p* < 0.05) in moisture content was observed between the pure MOLP and SDMOLPs, however, no significant difference was noted among the solid dispersions. The observed relatively low moisture content in the solid dispersions (SDMOLP) could be attributed to the effect of processing techniques and the added drying stage either by an oven, microwave, or freeze-drying, which removed the excess moisture. Lower moisture content is desirable in food powders as it indicates lower microbial activity and long storage life. 

#### 3.1.2. Protein

The crude protein of MOLP was 28.53%. The proximate analysis showed that MOLP is highly rich in protein. The observed high protein content in MOLP was in accordance with the previous findings of 28.00% by Bamishaiye et al. [3] and Okiki et al. [37], and comparable to the reported value of 28.9 g/100 g [38]. The result was, however, lower than 32.18%, as reported by Moyo et al. [39], but significantly higher than 22.67% and 24.20% as previously reported by Ali et al. [4,7]. The findings indicate that MOLP is a good source of protein. The differences in protein values from those reported in previous studies may be due to agro-climatic conditions during cultivation. The nutrient content of the plant varies with climate, soil, and other environmental conditions. Moreover, the variances are augmented by processing and storage.

Protein contents for solid dispersions (SDMOLP) ranged from 12.43 to 14.23%, with the highest value recorded for the melting solid dispersion (PEG6000 and PEG4000) The significant decrease observed in the protein of solid dispersions compared to MOLP could be attributed to processing or preparation conditions such as heat or carrier coating. Devisetti et al. [38] also reported a low protein content for pre-treated MOLP. However, it needs to be noted that the protein content of solid dispersions was still higher than the one for leafy vegetables (0.53–3.52%) [40]. It is worth noting that plant foods that contain more than 12% of their calorific content from proteins have been proven to be a healthy source of proteins [41]. This shows that all of the solid dispersions studied are high in protein and could play an important role in delivering affordable and readily available protein to rural areas. Moreover, the results suggest that solid dispersions can be used to develop high-protein beverages and foods. Appreciable protein content in MOLP and SDMOLPs makes them good sources of supplementary protein for man and livestock. MOLP constitutes a good source of protein in regions where animal protein is rare. Therefore, it is used to combat malnutrition concerning protein deficiency.

#### 3.1.3. Ash

The ash content is generally recognized as a measure of quality to assess the functional properties of foods [42]. The ash content of MOLP was 11.88%. The result is in good agreement with the results (11.60%, 12.0%) obtained by Okiki et al. [37]. Other earlier findings had reported a slightly lower ash content of MOLP that ranged between 8.05 and 10.38% [43]. In line with prior research, the results showed that MOLP contains high mineral concentrations. The high ash content of MOLP reflects the mineral content preserved in the food material. Therefore, the results suggest a high deposit of mineral elements in the leaves, further indicating that MOLP could be a good source of minerals, as previously reported in the literature. Variations in MOLP ash content might be attributed to differences in location, variety, environmental/soil type, and the temperature at which the leaves were dried. On the other hand, SDMOLPs exhibited ash contents that ranged from 4.03 to 6.16%. The maximum value was recorded for the PEG6000 solvent evaporation solid dispersion, whereas the lowest was obtained for the PEG4000 solvent evaporation dispersions. There was no substantial difference in the ash content amongst the SDMOLPs. However, a significant difference (*p* < 0.05) between the MOLP and SDMOLPs was observed. The ash contents of SDMOLPs were similar and comparable to the soybean values of 5.60% or cornmeal [44]. Ash in food contributes to the residue that remains after all of the moisture has been removed and organic components (fat, vitamins, protein, organic acid, carbohydrates, etc.) have been incinerated. The ash content thus determines a measure of mineral concentration in the dried leaf powder. In line with prior research, the results show that MOLP and SDMOLPs contain high mineral concentrations and are good sources of mineral elements. 

#### 3.1.4. Fat

The fat content of MOLP was 5.13%. This result agreed with previous results that reported fat values ranging from 4.03 to 9.51% [6]. This observation was also similar to the findings of other investigators who reported the fat content of MOLP as 4.00% [37]. Moreover, the MOLP fat content obtained in this study was comparable to that of Kenaf leaves (5.70%), as reported by Yee et al. [4,45]. The fat content obtained in this study was moderate when compared to those from other green leafy vegetables such as *Cardiospermum halicacabum*, *Pisonia grandis*, *Premna latifolia*, *Argyreia pomacea*, *Mollugo pentaphylla*, and *Delonix elata* (2.18–4.75%) [42]. On the other hand, the fat content of SDMOLPs ranged from 2.43 to 3.15%. The highest fat content was observed for the freeze-dried solid dispersions (both PEG4000 and PEG6000), whereas the lowest values were observed for the solvent evaporation dispersions (both PEG6000 and PEG4000). There was a significant difference between the solid dispersions. However, a significant difference existed between MOLP and the solid dispersions. The low-fat content of SDMOLPs was similar to the values (2.18–4.75%) for leafy vegetables such as *Cardiospermum halicacabum*, *Premna latifolia*, *Delonix elata*, *Argyreia pomacea*, *Mollugo pentaphylla*, and *Pisonia grandis* [42]. However, the fat contents of SDMOLP observed in this study were comparatively higher than the 0.38–1.91% reported for sweet potato leaves [46]. Low fat is desirable in foods as it plays a significant role in avoiding obesity. Fat in food determines the amount of energy available. Accordingly, Okiki et al. [37] postulated that a diet containing 1–2% of its calorific energy as fat is thought to be enough for humans, as high-fat consumption has been linked to various cardiovascular illnesses such as cancer and aging. Furthermore, *Moringa oleifera* leaves have a higher concentration of polyunsaturated fatty acids (PUFAs) than saturated fatty acids (SFAs). A higher PUFA content and a lower SFA content are desirable; hence, PUFAs should be included in the diet. They can help to prevent diseases and so promote excellent health [6]. Therefore, the consumption of these solid dispersions and MOLP in a large amount may be recommended for individuals suffering from obesity. 

#### 3.1.5. Carbohydrates

Carbohydrates are valuable components, which are the primary energy sources for the human body. The carbohydrate content for MOLP was calculated as 46.10%. MOLP is high in carbohydrates and has a high caloric value, which can help to meet the body’s caloric needs. Carbohydrates are an important part of a balanced and healthy diet, accounting for half of our daily calorie intake. This carbohydrate value of MOLP is similar to those (47.25 and 48.97%) reported by Sultana [6], and comparable to the 54.61 and 57.61% obtained by Valdez-Solana et al. [47]. The slight variations in carbohydrates of the MOLP obtained in the study and others could be explained through different soil types and organic matter. The carbohydrate content was in the range of 73.16–77.20% among the solid dispersions. The total carbohydrate content was highest in the solvent evaporation (PEG4000) solid dispersions while lowest in the melting (PEG4000) solid dispersions. There was no significant difference in the carbohydrates among the solid dispersions. These values are comparable to the ones reported for sweet potato leaves (82.8%) [41]. The results obtained in this study were also comparable with previously reported values of cassava (Chila cultivar), which ranged between 84.32 and 86.57% [48]. A significant increase (*p* < 0.05) in the carbohydrate content of solid dispersions with a corresponding decrease in the protein and fat contents was observed. The carbohydrate content was associated adversely with protein, fat, fiber, ash, and moisture content. The negative correlation showed that the key components influencing carbs were protein, fat, and moisture content and that a decrease in these molecules would result in a considerable increase in the total carbohydrates. The carbohydrate content of SDMOLP was significantly higher than that of MOLP. Carbohydrates are the principal sources of energy. One of the main functions of the soluble carbohydrate in the body is for energy supply, thus MOLP can be eaten as a vegetable or fortified in foods. The carbohydrate content of SDMOLP is high, therefore suggesting that it could be a good supplement as well as a source of energy. Generally, carbohydrates play an essential role in the bulk of diets as they provide energy to cells such as the brain, muscles, and blood [42].

The energy value of MOLP was 344.46 Kcal/100 g, while that of SDMOLP was in the range of 374.89–404.52 Kcal/100 g. The energy values obtained in this study were similar to the previously reported values of 353.03–368.17 Kcal/100 g for MOLP [6]. The energy contents observed in SDMOLPs were relatively (*p* < 0.05) higher than that of MOLP. The slight differences could be due to differences in the carbohydrate content. The high energy content in MOLP and SDMOLPs can contribute significantly to the daily caloric requirement of the body

### 3.2. Elemental Composition of MOLP and Solid-Dispersed MOLP

**Figure 1:**
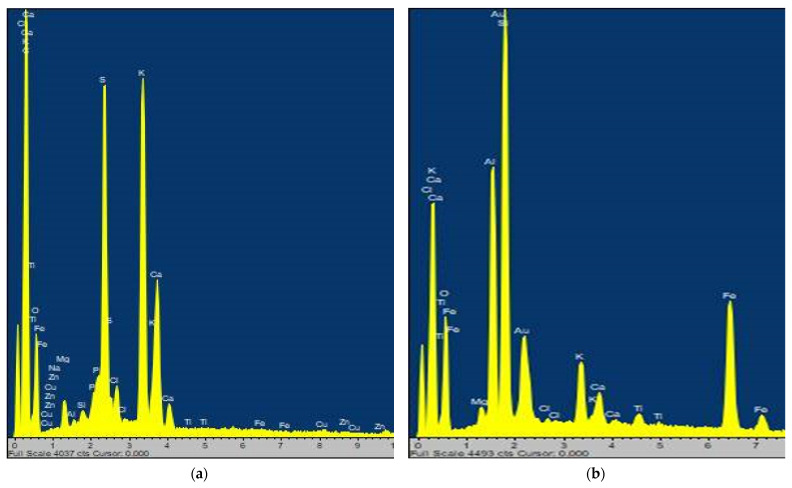
The SEM-EDS spectra of (**a**) MOLP and (**b**) SDMOLP.

Scanning electron micrography-energy-dispersive X-ray spectroscopy (SEM-EDX) is a characterization technique that provides the elemental composition of various constituent elements in a material. The SEM-EDX technique determines the distribution of various elements on the surfaces. Table 2 illustrates the elemental composition of the MOLP and SDMOLPs. The EDX spectrum of MOLP is shown in Figure 1. The results of the mineral composition of MOLP, as shown in Table 2, exhibited a high concentration of Ca (40.56%), K (8.29%), Mg (16.02%), P (13.71%), S (21.32%), Fe (6.09%), Cu (4.93%), and Zn (0.40%). While the elemental composition of solid dispersions ranged from 38.28 to 65.05% Ca, 4.24–17.97% Mg, 2.22–13.52% P, 0.15–6.09% Fe, 6.00–36.98% S, 0.00–8.49% Zn, and 0.00–2.93% Na. The elemental composition of the pure MOLP and SDMOLPs revealed that they are high in biologically significant elements with therapeutic properties, suggesting that they could supplement macro- and microelements in the body. The results obtained in this study were similar to those obtained by Raju et al. [49] using the same technique for MOLP, which exhibited 76.70% Ca, 2.53% Cu, 15.64% Zn, and 5.03% Mg. However, slight variation in the mineral composition may be explained by the difference in soil, weather, and maturity of the plant leaves, since the macronutrients of the plants might vary according to the biotic and abiotic conditions of the environment as well as the maturity of the leaves [50]. The MOLP mineral results also complement previous studies where MOLP is a source of macro and microelements. The elemental composition of solid dispersions revealed a higher concentration of calcium (varying from 46.87 to 65.05%) than MOLP (40.56%), with the highest value recorded for the melting method solid dispersions. Moreover, the solid dispersions had high concentrations of Mg of 17.97% (melting method), Cu of 5.33–27.05% (solvent evaporation method, microwave method, and melting method), S of 22.22–36.98% (microwave method, and freeze-dried method); and Zn of 0.66–8.49% (solvent evaporation method, and microwave method). The high elemental contents in solid dispersions suggest that they can be used as functional ingredients in foods or nutraceutical formulations or be taken as supplements. MOLP solid dispersions are a major source of minerals, and thus can be used to alleviate many mineral deficiencies (Ca, Mg, Cu, Zn). Consumers use solid dispersions in beverages, cereals, medicine, salads, and sauces to increase their nutritional density. Variations in the elemental composition among the solid dispersions can also be due to the different carriers and processing conditions [51].

The minerals found in *Moringa oleifera* leaves and solid dispersions may play a curative and preventive role in combating human disease. For example, calcium, as a macroelement, is a multifunctional nutrient essential to the body’s metabolism, and calcium deficiency leads to osteoporosis. Therefore, *Moringa oleifera* leaf powder is known as a natural remedy for osteoporosis. Calcium and phosphorous-containing substances are required by children, pregnant, and lactating women for the development of the bones and teeth. Furthermore, magnesium intake is good for diabetes [47]. Iron is required for the formation of hemoglobin and its deficiency leads to anemia. Therefore, MOLP and SDMOLPs can be taken as iron supplements. The elemental composition of the MOLP revealed low contents of zinc. This finding is in agreement with several studies that have reported low zinc content and no zinc [52]. Some elements including titanium, silicon iron, and chlorine were detected in small concentrations, as evidenced by the smaller EDX peaks and low intensity. Compared to the daily requirement of elementals, Moringa leaves and SDMOLPs are a good dietary source of calcium, sulfur, phosphorous, and magnesium, and one microelement: copper.

### 3.3. Functional Properties of MOLP and SDMOLPs

#### 3.3.1. Water Absorption Capacity

**Figure 2:**
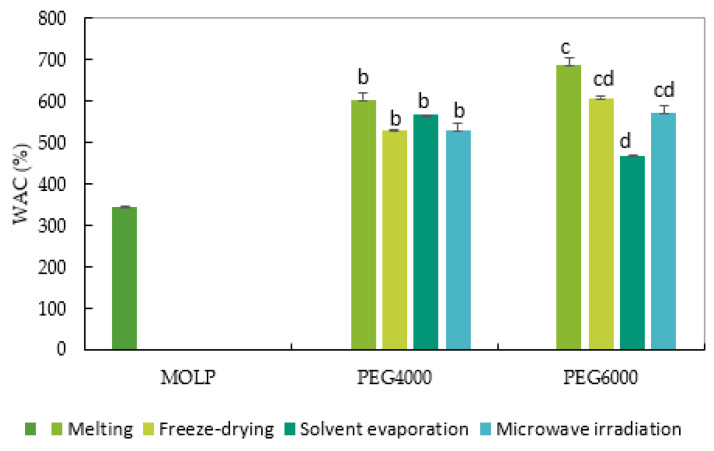
The water absorption capacity of MOLP and solid-dispersed MOLP prepared by melting, freeze-drying, solvent evaporation, and microwave methods using PEG4000 and PEG600 carriers. Bars with different superscripts in the same category (PEG) were significantly different (*p* < 0.05).

The water absorption capacity (WAC) of MOLP and solid dispersions is displayed in Figure 2. MOLP exhibited a high WAC of 345.09%, due to the presence of higher amounts of protein, carbohydrates, and dietary fiber in the leaves. The MOLP’s WAC concurs with the values previously reported (335%, 158.00%, 415%, 255.50%, and 350%) [6]. Devisetti et al. [38] reported a slightly higher WAC value (434.3 mg/100 g) for MOLP than the one obtained in this study. Interactions of protein with water are essential to properties such as hydration and solubility. The WAC is considered as a necessary attribute of food ingredients for formulating various value-added food products including bakery products and beverages [53]. On the other hand, the WAC of solid-dispersed MOLPs (SDMOLPs) ranged from 468.86% (solvent evaporation with PEG6000) to 686.37% (melting method with PEG6000). There was no statistically significant difference in the solid dispersions made with PEG4000 carriers. In contrast, a substantial difference was seen between the melting and solvent evaporation solid dispersions made with PEG6000. The SDMOLP formed by melting (PEG 6000 and PEG40000, respectively) had significantly greater WACs than the solid dispersions prepared by solvent evaporation and microwave. Freeze-dried SDMOLPs, on the other hand, showed a considerably greater WAC than solvent evaporation (PEG6000) and microwave dried SDMOLPs (PEG4000) The slight variances in WACs between the eight solid dispersions could be attributable to structural differences, the degrees of availability of water binding sites among the powders, and variations in the protein, carbohydrates, or fiber compositions due to different production methods. Agamou et al. [51] further noted that increased WAC could be linked to higher levels of compounds with hydrophilic characteristics such as total fibers in the leaves. Indeed, the leaves of *Moringa oleifera* are composed of cellulose, hemicellulose, and lignin, which are distinguished by polysaccharide chains capable of retaining water molecules via hydrogen bonds. Tafu and Jideani [22] reported that the presence of hydrogen bonds, molecular interactions between MOLP, and hydrophilic carriers, in the solid dispersions were the primary causes of the rise in their solubility, WACm and dissolution rate. Similar observations were reported by Oyeyinka [54]. All of the SDMOLPs had significantly higher WAC than the pure MOLP. The highest WAC of the SDMOLPs could be attributed to the presence of the higher amount of carbohydrates in them. A high WAC is associated with the better reconstitution abilities of powder [55]. The WAC of the powders and flours represents their ability to be associated with water (reconstitute) under limited water conditions. The WAC is therefore important for the quality and texture of food products because it stabilizes the food against undesirable effects.

#### 3.3.2. Solubility

**Figure 3:**
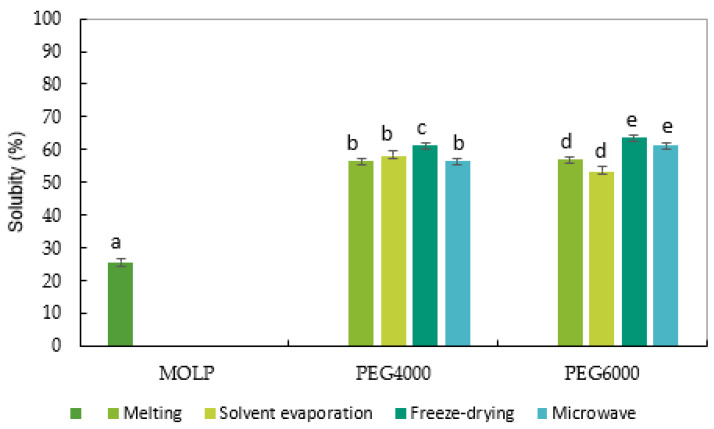
The solubility of the MOLP and solid-dispersed MOLPs prepared through melting, solvent evaporation, freeze-drying, and microwave irradiation using the PEG4000 and PEG6000 carriers. Bars with different superscripts in the same category (PEG) were significantly different (*p* < 0.05).

Solubility is the most reliable criterion for evaluating powders in an aqueous solution. Figure 3 shows the solubility of the pure MOLP and solid-dispersed MOLPs. The solubility of MOLP was 25.64%. The low solubility of MOLP agrees with those reported (25.3 mg/100 g) by Ali et al. [4] and Devisetti et al. [38], stating that MOLP has poor solubility and dissolution. The low solubility of MOLP can be attributed to the texture of the powder, which was observed to be fluffy, and less dense, thus making it hard to dissolve in water. We observed that the protein content directly affected the functional properties of powders. Indeed, the increase in the protein content contributed to reducing the WAC and solubility of the powders. The proteins are molecles that possess hydrophilic and lipophilic properties, and this result indicates that many factors limit the effect of these properties. Amongst those factors, is the presence of high anti-nutrient contents that chelate the proteins and limit their solubility [51]. In addition, the high protein, minerals, vitamins, and phenolic compounds found in MOLP may also account for its low solubility. On the other hand, the solubility of solid dispersed MOLPs varied significantly between 55% and 64%. The solid dispersion of PE6000 prepared by the freeze-drying method exhibited the highest solubility among all of the solid dispersions with a solubility reading of 64%, which was approximately twice as much as that of pure MOLP.

There was a considerable difference in the solubility of the PEG4000 solid dispersions between the melting and solvent evaporation solid dispersions, but no difference was found between the freeze-dried and microwave solid dispersions. In the PEG6000 solid dispersion, there was no significant difference between the melting and freeze-dried solid dispersions, nor between the microwave and solvent evaporation solid dispersions. Melting and freeze-dried solid dispersions, on the other hand, differed greatly from microwave and solvent evaporation solid dispersions. Compared to the MOLP, all of the solid dispersions demonstrated significantly improved (*p* < 0.05) solubility. The superior solubility profile observed for all dispersions was attributed to the amorphization of *Moringa oleifera* leaf powder by solid dispersion techniques, and the wetting characteristics of the hydrophilic carriers (PEG4000 and PEG6000) with MOLP. This result is in agreement with the studies reported by Newa et al. [56], Zawar and Bari [21], and Kumavat et al. [57], where they concluded that the solubility of ibuprofen, *Repaglinide*, *Curcumin,* respectively, increased when PEGs were added during solid dispersion processing using freeze-drying, melting, solvent evaporation, and microwave irradiation methods. PEG is a hydrophilic polymer used in many biochemical and industrial applications such as food, cosmetics, and pharmaceutical products. It is soluble in most solvents, thus resulting in solid dispersions that are highly soluble. Kumavat et al. [57] also confirmed that PEG as a carrier and coating agent increased the solubility of *Curcumin* solid dispersions. Therefore, the enhanced solubility in SDMOLPs might be attributed to the improved wetting in the presence of PEG4000 and PEG6000, and/or the intermolecular hydrogen bonding interactions between the functional groups of PEGs and MOLP (O–H, C–H, and N–H), and changes in the hydrophobic and van der Waals forces between MOLP and the hydrophilic carriers. Moreover, the increased solubility of SDMOLPs could be due to the particle size reduction, the solubilizing effect of the hydrophilic carriers, and the formation of soluble complexes between the hydrophilic carriers and poorly water-soluble MOLP [58]. As this is the first study to report on the solubility of Moringa solid dispersions, there are no previous findings.

## 4. Conclusions

This study demonstrated the feasibility of using PEG4000 and PEG6000 as hydrophilic carriers in the development of solid dispersions of MOLP. The study successfully employed the melting method, solvent evaporation method, freeze-drying method, and microwave irradiation method with PEG4000 and PEG6000 carriers to generate the solid dispersion for the solubility enhancement of MOLP. The functional properties (solubility and WAC) of the solid-dispersed MOLPs were significantly improved, relative to that of the pure MOLP. The enhancement of solubility from solid dispersions can be attributed to several factors such as better wetting in the presence of hydrophilic carriers, formation of hydrogen bonds between the functional groups of MOLP and carriers, particle size reduction, and particle porosity.Many plant ingredients’ bioavailability is compromised due to solubility issues, and thus solubility enhancement is required. Therefore, it is hoped that the new solid dispersions of MOLP with an improved solubility and WAC would be a promising solution to expand the application of MOLP in functional foods, beverages, and nutraceutical formulations. Furthermore, biochemical information on the nutritional and mineral compositions suggests that SDMOLPs are good sources of macro- and micronutrients and that they will contribute greatly toward meeting the nutritional requirements for normal growth while providing adequate protection against diseases arising from malnutrition.

## Figures and Tables

**Table 1:** The proximate compositions of pure *Moringa oleifera* leaf powder and solid dispersions of MOLP.

Samples	Moisture (%)	Protein (%)	Ash (%)	Fat (%)	CHO (%)	Energy (Kcal)
Pure MOLP	8.44 ± 1.60 ^a^	28.53 ± 1.61 ^a^	11.81 ± 1.20 ^a^	4.89 ± 0.80 ^a^	46.33 ± 2.45 ^a^	344.46 ± 9.22 ^a^
PEG4000						
Freeze-drying	4.03 ± 0.31 ^b^	12.4 ± 0.30 ^b^	5.48 ± 0.06 ^b^	3.15 ± 0.00 ^ab^	74.91 ± 0.28 ^b^	377.45 ± 1.94 ^ab^
Melting	3.56 ± 2.92 ^b^	14.2 ± 1.62 ^b^	6.14 ± 0.01 ^b^	2.85 ± 0.00 ^b^	73.22 ± 3.23 ^b^	404.52 ± 0.62 ^b^
Solvent evaporation	3.28 ± 0.83 ^b^	12.98 ± 0.81 ^b^	4.03 ± 0.20 ^b^	2.43 ± 0.11 ^b^	75.41 ± 1.10 ^b^	386.57 ± 8.10 ^ab^
Microwave irradiation	2.73 ± 1.10 ^b^	12.90 ± 1.11 ^b^	5.82 ± 0.61 ^b^	2.59 ± 0.00 ^b^	75.96 ± 0.49 ^b^	378.11 ± 3.00 ^ab^
PEG6000						
Freeze-drying	3.95 ± 0.63 ^b^	12.70 ± 0.61 ^b^	5.72 ± 0.09 ^b^	2.95 ± 0.00 ^b^	74.68 ± 0.40 ^b^	375.1 ± 2.06 ^ab^
Melting	3.07 ± 0.63 ^b^	13.23 ± 0.61 ^b^	5.66 ± 0.02 ^b^	2.84 ± 0.10 ^b^	75.30 ± 2.42 ^b^	374.89 ± 7.27 ^ab^
Solvent evaporation	2.65 ± 1.02 ^b^	12.9 ± 1.02 ^b^	6.16 ± 0.06 ^b^	2.27 ± 0.00 ^b^	76.00 ± 0.51 ^b^	375.53 ± 1.32 ^ab^
Microwave irradiation	3.10 ± 0.00 ^b^	12.52 ± 0.00 ^b^	5.89 ± 0.21 ^b^	2.75 ± 0.00 ^b^	75.72 ± 0.10 ^b^	377.66 ± 1.41 ^ab^

Values are expressed as the mean ± standard deviation. Means in a column not sharing the same letter are significantly different (*p* < 0.05). MOLP: *Moringa oleifera* leaf powder. PEG: Polyethylene glycol. CHO: Carbohydrate.

**Table 2:** The elemental composition of the MOLP and solid-dispersed MOLPs.

**Sample**	Ca	Mg	P	Fe	Cu	S	Zn	Na
MOLP	40.56 ± 6.1 ^a^	16.02 ± 7.6 ^a^	13.71 ± 1.13 ^a^	6.09 ± 4.78 ^a^	4.93 ± 0.11 ^a^	21.32 ± 2.38 ^a^	0.40 ± 0.08 ^a^	3.47 ± 2.70 ^a^
PEG4000								
Freeze-D	45.84 ± 3.8 ^a^	6.69 ± 9.1 ^b^	2.22 ± 0.66 ^b^	1.56 ± 2.20 ^b^	6.70 ± 7.83 ^a^	36.98 ± 23.0 ^b^	0.00 ± 0.00 ^a^	0.00 ± 0.00 ^a^
SE	46.87 ± 1.0 ^a^	8.92 ± 2.0 ^c^	13.52 ± 3.41 ^a^	3.75 ± 3.87 ^b^	7.2 ± 1.79 ^a^	17.63 ± 0.4 ^a^	1.48 ± 2.08 ^a^	0.56 ± 0.78 ^a^
Melting	65.05 ± 4.5 ^c^	11.53 ± 10 ^c^	3.79 ± 3.41 ^b^	0.15 ± 0.21 ^b^	5.33 ± 3.67 ^a^	13.65 ± 0.21 ^d^	0.03 ± 0.04 ^a^	0.48 ± 0.67 ^a^
Microwave	55.96 ± 2.6 ^d^	6.04 ± 3.5 ^b,c^	7.20 ± 2.44 ^c^	2.13 ± 3.01 ^b^	2.97 ± 4.20 ^a^	22.22 ± 0.51 ^a^	3.26 ± 4.6 ^ab^	0.28 ± 0.39 ^a^
PEG6000								
Freeze-D	64.83 ± 9.1 ^b^	4.24 ± 3.6 ^b^	1.73 ± 1.05 ^b^	3.20 ± 4.53 ^b^	5.65 ± 7.02 ^a^	18.64 ± 4.74 ^a^	0.34 ± 0.47 ^a^	1.40 ± 1.97 ^a^
SE	38.28 ± 1.5 ^a^	4.26 ± 6.0 ^b^	2.77 ± 3.91 ^b^	3.88 ± 0.19 ^b^	27.05 ± 25 ^b^	18.33 ± 0.35 ^a^	0.66± 0.93 ^a^	0.67 ± 0.95 ^a^
Melting	60.42 ± 5.0 ^c^	17.97 ± 1.4 ^a^	11.16 ± 8.51 ^a^	1.45 ± 1.89 ^b^	2.33 ± 0.82 ^a^	6.00 ± 1.07 ^c^	0.00 ± 0.00 ^a^	0.68 ± 0.95 ^a^
Microwave	38.31 ± 5.4 ^a^	10.02 ± 3.3 ^c^	6.61 ± 0.42 ^c^	2.53± 2.76 ^b^	7.00 ± 3.76 ^a^	24.14 ± 5.58 ^a^	8.49 ± 4.72 ^b^	2.93 ± 3.94 ^a^

Values are expressed as the mean ± standard deviation. Means in a column not sharing the same letter were significantly different (*p* < 0.05). MOLP: *Moringa oleifera* leaf powder, Freeze-D: freeze-drying, SE: solvent evaporation, PEG: polyethylene glycol, Ca: calcium; Mg: magnesium; S: sulfur; P: phosphorus; Fe: iron; Zn: zinc; Na: sodium.

## Data Availability

There are no data outside that reported in this article.

## Sample Availability

Samples of the compounds are not available from the authors.
